# Studies on the Exergy Transfer Law for the Irreversible Process in the Waxy Crude Oil Pipeline Transportation

**DOI:** 10.3390/e20050309

**Published:** 2018-04-24

**Authors:** Qinglin Cheng, Anbo Zheng, Shuang Song, Hao Wu, Lili Lv, Yang Liu

**Affiliations:** 1 Key Lab of Ministry of Education for Enhancing the Oil and Gas Recovery Ratio, Northeast Petroleum University, Daqing 163318, China; 2 PetroChina Planning & Engineering Institute (CPPEI), Daqing 163318, China

**Keywords:** non-equilibrium thermodynamics, entropy generation rate equation, entropy flow, exergy transfer equation, exergy flow transfer law

## Abstract

With the increasing demand of oil products in China, the energy consumption of pipeline operation will continue to rise greatly, as well as the cost of oil transportation. In the field of practical engineering, saving energy, reducing energy consumption and adapting to the international oil situation are the development trends and represent difficult problems. Based on the basic principle of non-equilibrium thermodynamics, this paper derives the field equilibrium equations of non-equilibrium thermodynamic process for pipeline transportation. To seek the bilinear form of “force” and “flow” in the non-equilibrium thermodynamics of entropy generation rate, the oil pipeline exergy balance equation and the exergy transfer pipeline dynamic equation of the irreversibility were established. The exergy balance equation was applied to energy balance evaluation system, which makes the system more perfect. The exergy flow transfer law of the waxy oil pipeline were explored deeply from the directions of dynamic exergy, pressure exergy, thermal exergy and diffusion exergy. Taking an oil pipeline as an example, the influence factors of exergy transfer coefficient and exergy flow density were analyzed separately.

## 1. Introduction

The common energy analysis in practical engineering problems is carried out at the “quantity” level. However, this method does not explain the energy utilization and energy loss at the “quality” level. Thus, in the process of energy saving, it is important to consider the role of exergy fully and effectively. This is the embodiment of energy quality, which can reduce unnecessary exergy loss. Thus, for the analysis of the exergy conversation and the exergy transformation in the waxy crude oil pipeline transportation process [1], the essence of the energy consumption in the oil transportation process was clarified. It is of great practical significance to the optimization and energy saving of the pipe transmission process. It also means a lot to the scientific community and reasonable use of energy in petroleum enterprises [2].

With the deepening theoretical research of thermodynamics, the local equilibrium differential equations were originated in 1961 and established by Gaggioli. As for the proposal and study of exergy transfer, its concept had not been put forward explicitly, but the thought of the exergy transfer had been implied [3]. In 1985, Soma J. proposed exergy transfer concept for the first time, defined exergy transfer coefficient and deduced the exergy transfer equation in one-dimensional steady heat conduction process [4]. In 1992, Dunber W.R. decomposed the energy equation and exergy equation, and the relationships between transfer and conversion of energy and exergy in different forms were analyzed concretely [5]. 

As a measure of quality, exergy is part of energy, so energy transfer and conversion process must have the “quality”, i.e., exergy transfer and conversion. The study of exergy transfer and conversion can correctly explain the mechanism of irreversible process. The calculation of exergy loss is an important method to increase the efficient utilization of energy and improve the transfer process performance [6]. The exergy transfer concept was introduced into China in the beginning of the 1990s [7]. In 1993, an energy transfer process was proposed by Xiang Xinyao: the combination of exergy thermodynamic properties and energy transfer law. He explained some basic concepts involved in energy transfer processes. The “monomers” separated from the system were called the basic element of exergy transmission. The exergy transfer model was established, in which the total exergy can be decomposed into thermal exergy, potential exergy, chemical exergy and other forms of exergy transmission. Solution steps on engineering exergy problem were also provided. In 1998, Wang et al. [8,9] started from the universalized energy and exergy equation, established the dynamic equation of universalized energy and exergy transfer. The equation is analyzed with decomposition. It can also clarify the different forms of exergy transfer and conversion rules. In 2002, Qiao et al. [10,11,12,13] studied the exergy transfer in the heat conduction process both for steady or non-steady state of one and multiple dimensions. The mechanism of exergy transfer in the heat conduction process is revealed. In 2009, Liu et al. [14] clarified the relationship between process dynamics or process resistance and process rate, which is based on the establishment of exergy transfer phenomenological equation. The ascertainment of the exergy transfer coefficient is the key in exergy transfer problem [15].

Thus, the description of exergy balance equation, the understanding of the conversion relationship between exergy flows and the establishment of the exergy dynamic equation are all essential development trends in the waxy crude oil pipeline energy analysis. Studies on the influence factors of the different exergy transfer processes are an important theoretical step in energy saving factors of the pipeline process.

## 2. Field Equilibrium Equation and Entropy Generation Rate Equation

### 2.1. Field Equilibrium Equation

For non-equilibrium systems, we divide the research system into several subsystems, where the macro size is small enough and the micro size is large enough. Since the macro is small enough, the thermodynamic property in each subsystem is a local thermodynamic equilibrium system. It is assumed to be a local equilibrium hypothesis. The main task of the study is to clarify the laws of the continuous model in the physical system. Thus, the concept of continuity hypothesis is introduced, which has two meanings: (1) material points occupy the whole space with no gap distribution; and (2) in the deformation process, material maintains continuity, that is, under the continuity hypothesis, the object deformation amount can be expressed as a continuous function of the coordinate. In a continuous medium, a thermodynamic variable can be regarded as a continuous function of time and space. Therefore, the physical quantity has a definite value at any point and any time in the system, and the physical quantities are the continuous functions of time and space coordinates. According to the thermodynamic basis of the above non-equilibrium state, the general equilibrium equation is described [16]: 

Set *A* to represent an arbitrary variable, $a=\frac{dA}{dm}$ is the specific parameters for unit mass, then $A=\int_{V}\rho adV$. The changes of physical quantity *A* within a time interval d*t* can be expressed as $\frac{dA}{dt}=\frac{d}{dt}\int_{V}\rho adV$.

According to the local description of Euler equation:

(1) $$\frac{\partial \rho a}{\partial t}+\nabla \cdot J_{a}^{}-\varepsilon_{a}=0$$

Lagrange description in the body form is

(2) $$\frac{d\rho a}{dt}+\nabla \cdot J_{a}-\varepsilon_{a}=0$$

A comparative analysis between Eulerian Equation (1) and Lagrange description in Equation (2) is consistent and then it is applied to mass conservation equation, component mass conservation equation, energy conservation equation and entropy equilibrium equation.

(1) Mass conservation equation

For unit microelement dxdydz, the equilibrium relation equation is:

(3) $$\frac{\partial \rho }{\partial t}dxdydz=-\left[\frac{\partial }{\partial x}\left(\rho W_{x}\right)+\frac{\partial }{\partial y}\left(\rho W_{y}\right)+\frac{\partial }{\partial z}\left(\rho W_{z}\right)\right]dxdydz=\left(-\mathrm{div}\rho W\right)dxdydz$$

where *W* is the flow velocity. The mass conservation equation in the body form is also called continuity equation.

(4) $$\frac{\partial \rho }{\partial t}+\mathrm{div}\rho W=0$$

(2) Component mass conservation equation

For the chemical phenomena of control body, the mass of component *i* is not conserved. Thus, the equilibrium relation equation is:

(5) $$\frac{\partial \rho_{i}}{\partial t}=-\mathrm{div}\rho_{i}W-\mathrm{div}j_{i}+\sum_{k=1}^{l}\Lambda_{ik}j_{k}$$

After transformation,

(6) −ρT∑i=1nηidcidt=−∑i=1nηi(∑k=1lΛikjk−divji)T

That is, the body form is expressed as:

(7) $$\rho \frac{d\eta_{i}}{dt}=-\mathrm{div}j_{i}+r_{i}$$

(3) Momentum balance equation

Only considering the situation of mechanical momentum, the expression of momentum equilibrium equation is obtained.

(8) $$\rho \frac{dW}{dt}=-\mathrm{div}\pi +\sum_{i=1}^{n}\rho_{i}f_{i}$$

The compressive stress tensor π can be expressed as $\pi =\pi^{e}+\pi^{v}$. $\pi^{e}$ contains the thermodynamic elastic part, which describes the reversible momentum transfer due to the fluid pressure stress. The viscous part $\pi^{v}$ describes the irreversible momentum transfer due to viscosity, and the Newton fluid is expressed as:

(9) $$\pi =-2\mu {\left(\nabla V\right)}_{s}+\left(\frac{2}{3}\mu -\eta \right)\left(\nabla \cdot V\right)\delta$$

(4) Energy conservation equation

The momentum equilibrium equation is transformed as:

(10) $$\rho W\cdot \left(\frac{dW}{dt}\right)=-\left(\mathrm{Div}\pi \right)\cdot W+\sum \rho_{i}F\cdot W=-\mathrm{div}\left(\pi \cdot W\right)+\pi :\mathrm{Grad}W+\rho_{i}F_{i}\cdot W$$

After simplification,

(11) $$\rho W\cdot \left(\frac{dW}{dt}\right)=\frac{\partial }{\partial t}\left(\frac{1}{2}\rho W^{2}\right)+\mathrm{div}\left[\left(\frac{1}{2}\rho W^{2}\right)\cdot W\right]$$

The body equation is obtained, which is combined with Gibbs equation,

(12) $$\rho \frac{du}{dt}=-\mathrm{div}j_{q}-\pi :\mathrm{Grad}W+\sum_{i=1}^{n}F_{i}\cdot j_{i}$$

(5) Universal entropy equilibrium equation

After the derivation of field equilibrium equation, it is possible to determine the entropy equation in non-equilibrium thermodynamics. Generally, the system entropy change d*S* consists of two parts: entropy flow d*S_e_* supplied by the outside world and entropy generation d*S_i_* due to the irreversible process in the system.

(13) $$dS=dS_{e}+dS_{i}$$

The system total entropy can be expressed as:

(14) $$S=\int_{v}\rho sdV$$

The system entropy flow can be expressed as:

(15) $$\frac{dS_{e}}{dt}=-\int_{\Omega }j_{s,t}\cdot d\Omega$$

where $j_{s,t}$ is the entropy passing through the unit area at unit time.

Entropy generation in the system can be expressed as:

(16) $$\frac{dS_{i}}{dt}=\int_{v}\sigma dV$$

where, σ is the entropy generation in unit volume at unit time, that is, entropy generation rate.

Substituting Equations (15) and (16) into Equation (13):

(17) $$\int_{v}\left(\frac{\partial \rho S}{\partial t}+\nabla \cdot j_{s,t}-\sigma \right)dV=0$$

Because any selection for volume is establishment, it can be obtained.

(18) $$\frac{\partial \rho s}{\partial t}=-\nabla \cdot j_{s,t}+\sigma ,\;\mathrm{that}\;\mathrm{is},\;\rho \frac{ds}{dt}=-\nabla \cdot j_{s}+\sigma$$

where the entropy flow $j_{s}$ is the difference between the total entropy flow $j_{s,t}$ and the convective term ρsV.

### 2.2. Entropy Generation Rate Equation of Waxy Crude Oil

Based on observation and experience, we know that there are some phenomenological laws to describe irreversible processes, such as Fourier’s law describing heat conduction, Fick’s law describing diffusion, Newton’s law describing viscous flow and Ohm’s law describing conduction process. If the system is near equilibrium state, the flow “*j_i_*” and its coupling force “*X_j_*” satisfy the linear relationship [17,18].

(19) $$j_{i}=\sum_{j=1}^{n}L_{ij}X_{j}\;(L_{ij}\;\mathrm{is}\;\mathrm{Phenomenological}\;\mathrm{coefficient})$$

The mass conservation equation, the component mass conservation equation and the energy balance equation are brought into the Gibbs equation, and the entropy equilibrium equation is obtained.

(20) $$\begin{array}{l} \rho \frac{ds}{dt}=\left\{-\mathrm{div}\left[\left(j_{q}-\sum_{i=1}^{n}\mu_{i}j_{i}\right)/T\right]\right\}\\ +\left\{-\left[j_{q}\cdot -\mathrm{grad}\left(\frac{1}{T}\right)\right]-\left[\sum_{i=1}^{n}j_{i}\cdot \left(T\mathrm{grad}\frac{\mu_{i}}{T}-F_{i}\right)/T\right]-\left[\left(\pi -p1\right):\mathrm{Grad}W\right]/T-\left(\sum_{k=1}^{l}\Lambda_{ik}j_{k}/T\right)\right\}\\ =-\mathrm{div}j_{i}+\sigma \end{array}$$

Chemical reactions are generally not considered in the pipeline transportation process of wax deposition, then $\sigma_{\mathrm{chemical}}=0$. Therefore, the universal entropy rate equation can be written as:

(21) $$\begin{array}{l} \sigma =\left\{j_{q}|-\frac{\mathrm{grad}T}{T^{2}}\right\}+\sum_{i=1}^{n}\left[j_{i}|-\left\{\mathrm{grad}\left(\mu_{i}/T\right)-\frac{F_{i}}{T}\right\}\right]+\left[\left(\pi -p1\right)|-\frac{\mathrm{Grad}W}{T}\right]\\ =j_{\mathrm{heat}}\cdot X_{\mathrm{heat}}+j_{\mathrm{mass}}\cdot X_{\mathrm{mass}}+j_{\mathrm{momentum}}\cdot X_{\mathrm{momentum}} \end{array}$$

(1) The heat transfer entropy generation rate 

In the waxy crude oil pipeline transportation process, the heat transfer is mainly due to heat conduction, which is between the pipe wall and the wax deposit layer, and the forced convection heat transfer, which is between the pipeline internal crude oil and wax deposit layer. When heat transfer is solved in the pipe, it is necessary to consider the physical parameter viscosity, which is greatly affected by the temperature. Other parameters can be determined according to a reference temperature. Considering the forced convection heat transfer between crude oil and wax deposit layer in the pipeline, it can be expressed by Dittus–Boelter formula. The pipeline axial heat flux density can be expressed by Newton’s law.

(22) jq1=hΔT=0.023Ref0.8Prf0.3λlΔT

(23) $$j_{r2}=-\lambda \frac{\partial T}{\partial z}$$

The total entropy generation rate of heat diffusion is as follows:

(24) $$\sigma_{\mathrm{heat}}=\left(Nu_{f}\frac{\lambda }{l}\Delta T+\lambda \frac{\partial T}{\partial z}\right)\frac{\left(\frac{\partial T}{\partial r}+\frac{\partial T}{\partial r}\right)}{T^{2}}$$

(2) The viscous entropy generation rate 

The viscosity of the relative motion between particles that hinders the fluid flow can be expressed as the following by the Newtonian internal friction law in the laminar flow.

(25) $$\sigma_{\mathrm{laminar}}=\left[\tau |-\frac{\mathrm{grad}W}{T}\right]=\left(\frac{\Delta pr}{2l}\right)\frac{\left(\frac{\partial v_{z}}{\partial r}\right)}{T}$$

When the flow is turbulent, the stress τ can be written as: $\tau =\rho l^{2}{\left(\frac{\partial v}{\partial r}\right)}^{2}$, therein $\frac{\partial v}{\partial r}=\frac{1}{\mathrm{kr}}\sqrt{\frac{\tau }{\rho }}$.

The entropy generation rate is

(26) $$\sigma_{\mathrm{turbulent}}=\left[\rho l^{2}{\left(\frac{\partial v}{\partial r}\right)}^{2}\frac{1}{\mathrm{kr}}\sqrt{\frac{\tau }{\rho }}\right]$$

(3) The mass transfer entropy generation rate

The diffusion flow does not consider the pressure diffusion during the transport process, thus $J=-c\left(D^{M}\nabla x+D^{T}\nabla T\right)$. Only considering the radial diffusion in pipe transmission process, the radial molecular diffusion of liquid–solid two-phase mixtures can be written as follows:

(27) $$j_{r1}=-c_{j}D_{{}_{1,2}}^{M}\frac{\partial z_{i}}{\partial T}\frac{\partial T}{\partial r},\;\mathrm{therein}\;D_{1,2}=\frac{\kappa T}{6\pi r^{\prime }\mu_{B}}$$

Combined with the Wilson equation, the entropy generation rate equation after calculation and deformation is as follows:

(28) $$\sigma_{\mathrm{mass}}=\sum_{i=1}^{n}\left\{\left[-c\frac{\kappa T}{6\pi r^{\prime }\mu_{B}}\cdot \frac{\partial x_{i}}{\partial T}\cdot \frac{\partial T}{\partial r}\right]|\left[-\frac{R}{x_{A}x_{B}}\left(1+\frac{\partial \ln\gamma_{A}}{\partial x_{A}}\right)\right]\right\}$$

According to the results of above entropy generation rate calculation, the formula for the total entropy generation rate at laminar flow is as follows:

(29) $$\sigma_{\mathrm{total}}=\left(Nu_{f}\frac{\lambda }{l}\Delta T+\lambda \frac{\partial T}{\partial z}\right)\frac{\left(\frac{\partial T}{\partial r}+\frac{\partial T}{\partial r}\right)}{T^{2}}+\left[\left(\frac{\Delta pr}{2l}\right)\frac{\left(\frac{\partial v_{z}}{\partial r}\right)}{T}\right]+\sum_{i=1}^{n}\left\{\left[-c\frac{\kappa T}{6\pi r^{\prime }\mu_{B}}\cdot \frac{\partial x_{i}}{\partial T}\cdot \frac{\partial T}{\partial r}\right]|\left[-\frac{R}{x_{A}x_{B}}\left(1+\frac{\partial \ln\gamma_{A}}{\partial x_{A}}\right)\right]\right\}$$

The total entropy generation rate in turbulent flow is as follows:

(30) $$\sigma_{\mathrm{total}}=\left(Nu_{f}\frac{\lambda }{l}\Delta T+\lambda \frac{\partial T}{\partial z}\right)\frac{\left(\frac{\partial T}{\partial r}+\frac{\partial T}{\partial r}\right)}{T^{2}}+\left[\rho l^{2}{\left(\frac{\partial v}{\partial r}\right)}^{2}\frac{1}{\mathrm{kr}}\sqrt{\frac{\tau }{\rho }}\right]+\sum_{i=1}^{n}\left\{\left[-c\frac{\kappa T}{6\pi r^{\prime }\mu_{B}}\cdot \frac{\partial x_{i}}{\partial T}\cdot \frac{\partial T}{\partial r}\right]|\left[-\frac{R}{x_{A}x_{B}}\left(1+\frac{\partial \ln\gamma_{A}}{\partial x_{A}}\right)\right]\right\}$$

## 3. Waxy Crude Oil Pipeline Exergy Transfer Equation

### 3.1. The Establishment of Exergy Transfer Model

From above equilibrium equations, in general, the broad extension $x_{ij}$ of the unit mass system that describes the *j* component in the *i* energy form has the following relation:

(31) $$\rho \frac{dx_{ij}}{dt}=-\frac{\partial j_{ijk}}{\partial x_{k}}+g_{ij}$$

The strength amount $X_{ij}$ corresponding to the extension amount $x_{ij}$ is multiplied with the equation. Collated as:

(32) $$\rho \frac{d\left(X_{ij}x_{ij}\right)}{dt}=-\frac{\partial \left(j_{ijk}X_{ij}\right)}{\partial x_{k}}+j_{ijk}\frac{\partial X_{ij}}{\partial x_{k}}+X_{ij}g_{ij}+\rho x_{ij}\frac{dX_{ij}}{dt}$$

To evaluate different kinds of energy, the physical quantity “exergy” is proposed based on the first and second laws, which is defined as the part of energy that can be theoretically converted into useful work when the system has a reversible change to a dead state [19].

Energy for ordinary unit mass:

(33) $$e=\sum_{i=1}^{N+1}\sum_{j=1}^{m}X_{ij}x_{ij}-\sum_{i=1}^{N+1}\sum_{j=1}^{m}X_{ij0}x_{ij0}=\sum_{i=1}^{N+1}\sum_{j=1（i\neq j）}^{m}\left(X_{ij}-X_{ij,0}\right)x_{ij}$$

For the unit mass of exergy

(34) $$\rho \frac{dex}{dt}=-\nabla \cdot j^{ex}-d^{ex}+g^{ex}$$

where, $j^{ex}$ is the eexergy flow density; $d^{ex}$ is the exergy loss rate in the unit quality system; and $g^{ex}$ is the external force exergy rate for the unit mass system 

According to the Equations (32) and (34), the transformation is:

(35) $$\rho \frac{de_{x}}{dt}=-\nabla \cdot \left[J_{ijk}\left(X_{ij}-X_{ij0}\right)\right]+J_{ijk}\nabla X_{ij}+\left(X_{ij}-X_{ij0}\right)g_{ij}+\rho x_{ij}\frac{dX_{ij}}{dt}$$

### 3.2. The Exergy Dynamics Transfer Equation

The work process is always associated with the concept of imbalance. No matter what kind of work, refrigerant or system is changed from initial state to a final state. Exergy is defined as the energy which has the maximum theoretical work ability. The maximum theoretical work ability is the work ability from the given state to the environment phase balance through a reversible change process. Thus, exergy is a state parameter. Dynamic exergy, pressure exergy, chemical exergy and thermal exergy in the waxy crude oil pipeline are calculated separately, and the exergy balance equation is obtained.

(36) $$e_{x}=\frac{1}{2}V^{2}+\left(T-T_{0}\right)s-\left(p-p_{0}\right)v+\sum_{n=1}^{N}\left(\mu_{n}-\mu_{n,0}\right)\eta_{n}$$

For the actual fluid flow process, the viscous effect will cause the corresponding shear force generated between the fluid layers and produce the flow velocity gradient. The generated flow is dynamic exergy flow. Due to the existence of pressure difference of pipe between the oil flow and the ambient, the work capacity for crude oil pipeline system is the pressure exergy. For heated transport pipeline, since the crude oil temperature is much higher than that of the pipeline outer wall, there is a certain radial temperature difference. The axial pipeline temperature drop is caused by this difference, and the generated flow is thermal exergy flow. Due to the difference between each component chemical potential in the waxy oil pipeline and the ambient chemical condition potential, the work capacity is defined as diffusion exergy. The chemical potential gradient is the driving force which causes the system diffusion exergy transfer, and the generated flow is chemical exergy flow [20,21,22,23,24].

(1) The dynamic exergy transfer equation.

The work ability due to the momentum generated by the pipeline flow movement is dynamic exergy in the crude oil pipeline system.

(37) $$E_{v}=\frac{1}{2}V^{2}$$

The system dynamic exergy transfer equation can be obtained from the exergy transfer basic equations:

(38) $$\rho \frac{d}{dt}\left[\frac{V^{2}}{2}\right]=-\nabla \cdot \left(P^{\mathrm{sv}}\cdot v\right)-v\cdot \left(\nabla \cdot P_{n}\right)+{\left(P^{\mathrm{sv}}\right)}^{T}:\nabla v$$

The first item on the right side of the equation expresses the dynamic exergy that is input from external body element to internal body element under the action of the viscous shear force. The second item represents the part of dynamic exergy reversibly converted into pressure exergy under the action of orthonormal variable pressure. The third item expresses the part of dynamic exergy irreversibly transformed into thermal exergy under the action of the viscous shear force.

(2) The pressure exergy transfer equation

The work ability due to pressure difference between the oil flow and the external environment is pressure exergy in the crude oil pipeline system.

(39) $$E_{\mathrm{xp}}=\int -\left(p-p_{0}\right)dV$$

The system pressure exergy transfer equation can be obtained from the exergy transfer basic equations:

(40) $$\rho \frac{d}{dt}\left[-\left(p-p_{0}\right)V\right]=-\nabla \cdot \left[\left(P_{n}-p_{0}\delta \right)\cdot v\right]+v\cdot \left(\nabla \cdot P_{n}\right)+\left[\left(P_{nv}\right)\cdot \nabla v\right]$$

The first item on the right side of the equation expresses the pressure exergy which inputs the pipeline system affected by static pressure and fluid internal viscous stress. The second item represents the part of pressure exergy converted into dynamic exergy. The third item indicates the part of pressure exergy irreversibly transformed into thermal exergy due to oil flow viscosity.

(3) The thermal exergy transfer equation

The work ability due to temperature difference between the oil flow and the external environment is thermal exergy in the crude oil pipeline system.

(41) $$E_{\mathrm{xh}}=\int \left(T-T_{0}\right)ds$$

The system thermal exergy transfer equation can be got from the exergy transfer basic equations:

(42) $$\rho \frac{d}{dt}\left[\left(T-T_{0}\right)s\right]=-\left[\nabla \cdot \left(1-\frac{T_{0}}{T}\right)Tj_{s}\right]+\frac{T_{0}}{T}\left(j_{s}\cdot \nabla T\right)-\left(1-\frac{T_{0}}{T}\right)\left[\left(P_{nv}\right)\cdot \nabla v\right]-\left(1-\frac{T_{0}}{T}\right)\left(\sum_{n=1}^{M}j_{n}\cdot \nabla \mu_{n}\right)$$

The first item on the right side of the equation expresses the pressure exergy which inputs the pipeline system through thermal conduction, thermal convection and thermal radiation. The second item represents the part of irreversible demotion caused by the heat loss in the thermal exergy transfer process. The third item indicates the part of pressure exergy irreversibly converted into thermal exergy. The fourth item expresses the diffusion exergy irreversibly transformed into thermal exergy. 

(4) The diffusion exergy transfer equation

Chemical exergy due to the unbalanced concentration or composition is diffusion exergy, of which system and environment are under constrained equilibrium state.

(43) $$E_{\mathrm{xc}}=\sum_{n=1}^{M}\left(\mu_{n}-\mu_{n,0}\right)\eta_{n}$$

(44) $$\rho \frac{d}{dt}\left[\sum_{n=1}^{M}\left(\mu_{n}-\mu_{n,0}\right)\eta_{n}\right]=-\nabla \cdot \left[\sum_{n=1}^{M}\left(\mu_{n}-\mu_{n,0}\right)j_{n}\right]+\left(\sum_{n=1}^{M}j_{n}\cdot \nabla \mu_{n}+\sum_{n=1}^{M}r_{n}\cdot \mu_{n}\right)$$

The first item on the right side of Equation (44) expresses the diffusion exergy of the input system. The second item represents the part of pressure exergy irreversibly transformed into thermal exergy. 

Since there is no external force on work exergy rate, the exergy transfer equation can be expressed by the sum of Equations (38), (40), (42) and (44) [25]:

(45) $$\rho \frac{de_{x}}{dt}=-\nabla \cdot j^{ex}-d^{ex}$$

(46) $$j^{ex}=\left(1-\frac{T_{0}}{T}\right)Tj_{s}+\left(P-p_{0}\delta \right)\cdot V+P^{\mathrm{sv}}\cdot V+\sum_{n=1}^{N}\left(\mu_{n}-\mu_{n,0}\right)j_{n}$$

(47) $$d^{ex}=T_{0}\sigma_{\mathrm{total}}$$

## 4. Waxy Crude Oil Pipeline Exergy Transfer Law

According to Equation (19), when the exergy flow is affected by the potential field, $j_{i}=E_{x}{}_{i}$ is exergy flow, $X_{j}$ expresses the potential force and $L_{ij}=K_{ij}$ expresses the exergy flow phenomenological coefficient generated by a potential force. $E_{i}$ shows the exergy transfer amount per unit time and per unit area, namely the exergy flow density or exergy flux. Exergy transfer coefficient can be used to measure the substance exergy transfer ability, which is a physical quantity determined by the transmission exergy element properties and the structure of itself. It directly affects the distribution of exergy flow density, and exergy transfer coefficient is the characteristic sign of exergy transfer process.

In any exergy transfer process, the irreversibility in the process is the factors which influence the exergy transfer coefficient. The exergy transfer coefficient and exergy flow density in the pipeline transportation are calculated.

(1) The dynamic exergy transfer law.

The dynamic exergy is considered as a mechanical exergy. Kinetic energy is the theoretical work ability of the system caused by the velocity difference relative to the environmental datum. Therefore, the intensive quantity refers to the velocity, and the wide extension is the momentum. The velocity gradient causes the dynamic exergy transfer force, so the corresponding generated flow is called dynamic exergy flow.

According to the velocity gradient formula, the dynamic exergy transfer coefficient is:

(48) $$K_{ev}=\frac{2\tau }{-\mu V^{2}}$$

The dynamic exergy flow density is:

(49) $$E_{v}=\frac{2\tau \nabla V}{-\mu V^{2}}$$

(2) The pressure exergy transfer law.

Pressure difference is the power to drive the pressure exergy transfer in the system. Therefore, the intensive quantity of the process refers to the pressure *P*, and the wide extension is the medium volume *V*. The pressure gradient causes pressure exergy transfer force, so the generated flow is called pressure exergy flow.

According to the pressure drop formula, the pressure exergy transfer coefficient is:

(50) $$K_{ep}=\frac{8\left(P-P_{0}\right)V}{\pi d\lambda L\rho v^{2}}$$

The pressure exergy flow density is:

(51) $$E_{p}=\frac{8\left(P-P_{0}\right)V\nabla P}{\pi d\lambda L\rho v^{2}}$$

(3) The thermal exergy transfer law

Temperature difference is the driving force of the oil flow to transfer heat. Therefore, the intensive quantity of the process refers to temperature *T*, and the wide extension is entropy s. The temperature gradient causes thermal exergy transfer force, so the generated flow is called thermal exergy flow.

According to the temperature drop formula, the thermal exergy transfer coefficient is:

(52) $$K_{et}=\frac{4\left[mc_{p}\left(T-T_{0}\right)-mc_{p}T_{0}\cdot \ln\frac{T}{T_{0}}\right]}{\pi d^{2}\left(T_{R}-T_{0}\right)e^{-\frac{k\pi DL}{Gc_{p}}}}$$

The thermal exergy flow density is:

(53) $$E_{t}=\frac{4\left[mc_{p}\left(T-T_{0}\right)-mc_{p}T_{0}\cdot \ln\frac{T}{T_{0}}\right]\nabla T}{\pi d^{2}\left(T_{R}-T_{0}\right)e^{-\frac{k\pi DL}{Gc_{p}}}}$$

(4) The diffusion exergy transfer law

Chemical potential difference is the power to drive the diffusion exergy transfer. Therefore, the intensive quantity of the process refers to chemical potential *μ*, and the wide extension is mole number *ω_n_*. Chemical potential gradient causes diffusion exergy transfer, so the generated flow is called chemical exergy flow.

According to the wax molecular concentration gradient calculation formula, the diffusion exergy transfer coefficient is:

(54) $$K_{ec}=\frac{4RT_{0}}{\pi d^{2}}\frac{\rho_{L}D_{m}}{W_{ml}}\sum_{n=1}^{M}\omega_{i,0}\ln\frac{a_{i,0}}{a_{i}^{0}}$$

The diffusion exergy flow density is:

(55) $$E_{c}=\frac{4RT_{0}}{\pi d^{2}}\frac{\rho_{L}D_{m}}{W_{ml}}\sum_{n=1}^{M}\omega_{i,0}\ln\frac{a_{i,0}}{a_{i}^{0}}\nabla C$$

(5) The total exergy flow

Combining Equations (30)–(37) obtains the total flow:

(56) $$\begin{array}{l} j^{ex}=E_{v}+E_{p}+E_{t}+E_{c}=\frac{2\tau \nabla V}{-\mu V^{2}}+\frac{8\left(P-P_{0}\right)V\nabla P}{\pi d\lambda L\rho v^{2}}\\ +\frac{4\left[mc_{p}\left(T-T_{0}\right)-mc_{p}T_{0}\cdot \ln\frac{T}{T_{0}}\right]\nabla T}{\pi d^{2}\left(T_{R}-T_{0}\right)e^{-\frac{k\pi DL}{Gc_{p}}}}+\frac{4RT_{0}}{\pi d^{2}}\frac{\rho_{L}D_{m}}{W_{ml}}\sum_{n=1}^{M}\omega_{i,0}\ln\frac{a_{i,0}}{a_{i}^{0}}\nabla C \end{array}$$

(6) The exergy kinetic equation

According to Equation (28), with the analysis of entropy generation characteristics in the waxy crude oil pipeline, the total exergy kinetic equation of laminar flow and turbulent flow has been obtained.

When it comes to laminar flow:

(57) $$\begin{array}{ll} \rho \frac{de_{x}}{dt} & =-\nabla \cdot j^{ex}-d^{ex}\\ & =-\left(\frac{2\tau \nabla V}{-\mu V^{2}}+\frac{8\left(P-P_{0}\right)V\nabla P}{\pi d\lambda L\rho v^{2}}+\frac{4\left[mc_{p}\left(T-T_{0}\right)-mc_{p}T_{0}\cdot \ln\frac{T}{T_{0}}\right]\nabla T}{\pi d^{2}\left(T_{R}-T_{0}\right)e^{-\frac{k\pi DL}{Gc_{p}}}}+\frac{4RT_{0}}{\pi d^{2}}\frac{\rho_{L}D_{m}}{W_{ml}}\sum_{n=1}^{M}\omega_{i,0}\ln\frac{a_{i,0}}{a_{i}^{0}}\nabla C\right)\\ & =-T_{0}\left\{\begin{array}{l} \left(Nu_{f}\frac{\lambda }{l}\Delta T+\lambda \frac{\partial T}{\partial z}\right)\frac{\left(\frac{\partial T}{\partial r}+\frac{\partial T}{\partial r}\right)}{T^{2}}+\left[\left(\frac{\Delta pr}{2l}\right)\frac{\left(\frac{\partial v_{z}}{\partial r}\right)}{T}\right]\\ +\sum_{i=1}^{n}\left\{\left[-c\frac{\kappa T}{6\pi r^{\prime }\mu_{B}}\cdot \frac{\partial x_{i}}{\partial T}\cdot \frac{\partial T}{\partial r}\right]|\left[-\frac{R}{x_{A}x_{B}}\left(1+\frac{\partial \ln\gamma_{A}}{\partial x_{A}}\right)\right]\right\} \end{array}\right\} \end{array}$$

When it comes to turbulent flow:

(58) $$\begin{array}{ll} \rho \frac{de_{x}}{dt} & =-\nabla \cdot j^{ex}-d^{ex}\\ & \;=-\left(\frac{2\tau \nabla V}{-\mu V^{2}}+\frac{8\left(P-P_{0}\right)V\nabla P}{\pi d\lambda L\rho v^{2}}+\frac{4\left[mc_{p}\left(T-T_{0}\right)-mc_{p}T_{0}\cdot \ln\frac{T}{T_{0}}\right]\nabla T}{\pi d^{2}\left(T_{R}-T_{0}\right)e^{-\frac{k\pi DL}{Gc_{p}}}}+\frac{4RT_{0}}{\pi d^{2}}\frac{\rho_{L}D_{m}}{W_{ml}}\sum_{n=1}^{M}\omega_{i,0}\ln\frac{a_{i,0}}{a_{i}^{0}}\nabla C\right)\\ & \;=-T_{0}\left\{\begin{array}{l} \left(Nu_{f}\frac{\lambda }{l}\Delta T+\lambda \frac{\partial T}{\partial z}\right)\frac{\left(\frac{\partial T}{\partial r}+\frac{\partial T}{\partial r}\right)}{T^{2}}+\left[\rho l^{2}{\left(\frac{\partial v}{\partial r}\right)}^{2}\frac{1}{\mathrm{kr}}\sqrt{\frac{\tau }{\rho }}\right]\\ +\sum_{i=1}^{n}\left\{\left[-c\frac{\kappa T}{6\pi r^{\prime }\mu_{B}}\cdot \frac{\partial x_{i}}{\partial T}\cdot \frac{\partial T}{\partial r}\right]|\left[-\frac{R}{x_{A}x_{B}}\left(1+\frac{\partial \ln\gamma_{A}}{\partial x_{A}}\right)\right]\right\} \end{array}\right\} \end{array}$$

According to the general form of the field equilibrium equations (Equations (4), (7), (9) and (12)), the universal entropy equilibrium equation (Equations (15) and (20)) is obtained by the same theorem. Based on the relationship between the thermodynamic force and flow in non-equilibrium state, the different expression equations (Equations (24)–(30)) of waxy crude oil entropy generation rate are obtained. According to the description of the dead state, the exergy balance equation (Equation (33)) is established, obtaining the exergy transfer equation (Equation (35)). Exergy is divided into several angles such as dynamic exergy, pressure exergy, thermal exergy and diffusion exergy to describe the exergy balance equation (Equation (36)). Then, the transfer relationships for different exergy (Equations (38), (40), (42), (44)) are obtained, and the relationship of force and flow is applied to difference exergy (Equation (46)). According to the waxy crude oil characteristics, the exergy transfer coefficient and exergy flow density (Equations (48)–(55)) are obtained, and the total exergy kinetic equation is established combining the entropy generation rate equation (Equations (57) and (58)). 

As the decisive factors for the wax crude oil exergy transfer capacity are the exergy transfer coefficient and the exergy flow density, further analysis of the influence factors is beneficial to the subsequent research in reducing energy consumption in the pipeline process. A certain problem can be found and improved from specific factors.

## 5. Analysis of Influence Factors for Exergy Transfer in Waxy Crude Oil Pipeline Process

Various types of exergy flow density and exergy transfer coefficient were calculated and analyzed as an example of crude oil pipeline. The average velocity is 0.6137 m/s, and the calculation does not consider dynamic exergy. The basic properties of crude oil and the operating parameters in the pipeline are shown in Table 1.

### 5.1. Analysis of Pressure Exergy Transfer Influence Factors

**Figure 1:**
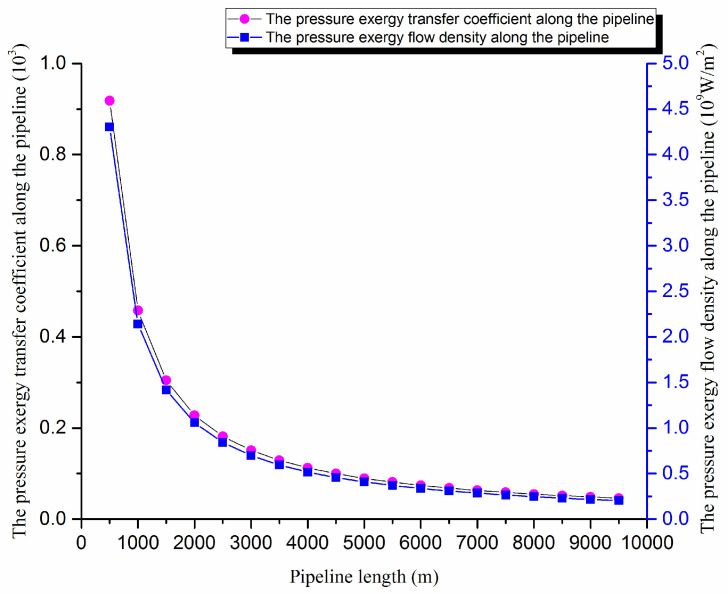
The pressure exergy transfer coefficient change curve and the pressure exergy flow density change curve of pipeline.

According to the crude oil basic physical properties and the operating parameters of pipeline, the pressure field, the pressure exergy transfer coefficient and the pressure exergy flow density are calculated using the above formula. The pressure exergy transfer coefficient change curve and the pressure exergy flow density change curve along the pipeline are shown in Figure 1:

The axial pressure field of pipe gradually decreases as the frictional resistance of pipe increases gradually. In Figure 1, the pressure exergy transfer coefficient and the pressure exergy flow density decrease, and they have large declines at the beginning of the transfer. In the transfer process, the decreasing amplitude decreases because the pressure difference which can push the pressure exergy transfer is great in the initial stage. With the advance of oil flow in the pipe, the pressure difference decreases, so the pressure exergy transfer strength and ability are lessened.

1. Pressure exergy transfer analysis in the pipeline transportation process under different outbound pressure conditions

**Figure 2:**
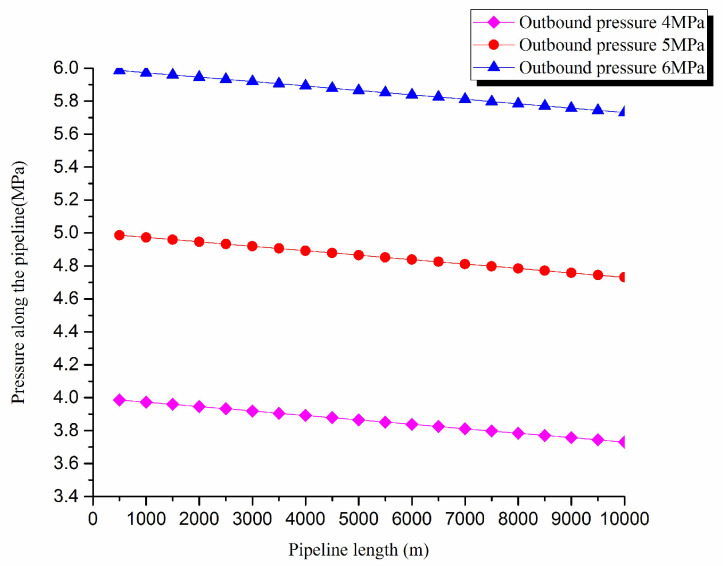
Pipe axial pressure field distribution curves under different outbound pressure conditions.

**Figure 3:**
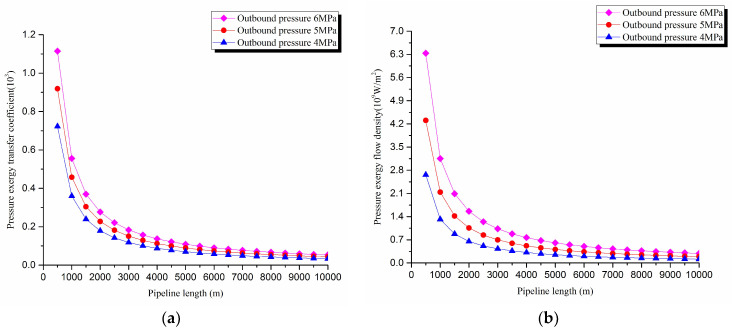
(**a**) Pressure exergy transfer coefficient change curves under different outbound pressure conditions. (**b**) Pressure exergy flow density change curves under different outbound pressure conditions.

The pressure exergy transfer law curves have been obtained under the working conditions of 4 MPa, 5 MPa, and 6 MPa in the pipeline transportation process, as shown in Figure 2 and Figure 3.

From the pressure exergy transfer conditions under different outbound pressures, it can be seen that the outbound pressure along the pipeline is higher, the pressure exergy transfer coefficient increases, and the pressure exergy flow density is greater. In the initial stage of pipeline, the outbound pressure has great influence on the pressure exergy transfer, the pressure exergy transfer coefficient and the pressure exergy flow density increase obviously, and the slope of the curve also becomes obviously greater. With the increase of transmission distance, the influence of the increasing outbound pressure on the pressure exergy transfer coefficient and the pressure exergy flow density decreases gradually. However, the overall change trend remains unchanged. It can explain the phenomenon that the increase of pressure can strengthen the pressure exergy transfer and the effect is most obvious at the beginning of the pipeline.

2. Pressure exergy transfer analysis in the pipeline transportation process under different flow conditions

**Figure 4:**
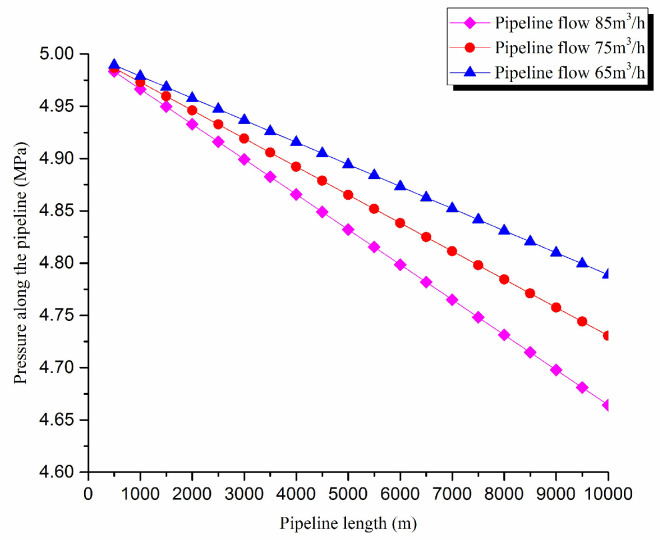
Pipe axial pressure field distribution curves under different flow conditions.

**Figure 5:**
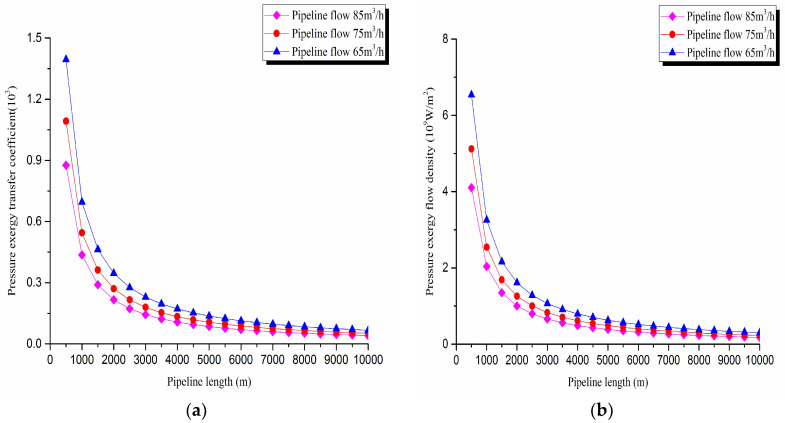
(**a**) Pressure exergy transfer coefficient change curves under different flow conditions. (**b**) Pressure exergy flow density change curves under different flow conditions.

The pressure exergy transfer law curves have been obtained under the working conditions of 65 m^3^/h, 75 m^3^/h, and 85 m^3^/h, as shown in Figure 4 and Figure 5.

As can be seen in these figures, when the pipe outbound pressure and the external environment pressure are certain, the greater is the flow of pipeline, the faster is the pressure drop in the pipeline. From the pressure exergy transfer conditions under different flow, it can be seen that the flow is higher, the pressure exergy transfer coefficient along the pipeline decreases, and the pressure exergy flow density lessens. In the initial stage of pipeline, the increase of flow has great influence on the pressure exergy transfer, the pressure exergy transfer coefficient and the pressure exergy flow density decrease evidently, and the slope of the curve is also decreasing. With the increase of transmission distance, the influence of the increasing flow on the pressure exergy transfer coefficient and the pressure exergy flow density decreases gradually. However, the overall change trend remains unchanged. It can explain the phenomenon that the increase of flow can weaken the pressure exergy transfer and the effect is also most obvious at the beginning of the pipeline.

### 5.2. Analysis of Thermal Exergy Transfer Influence Factors

**Figure 6:**
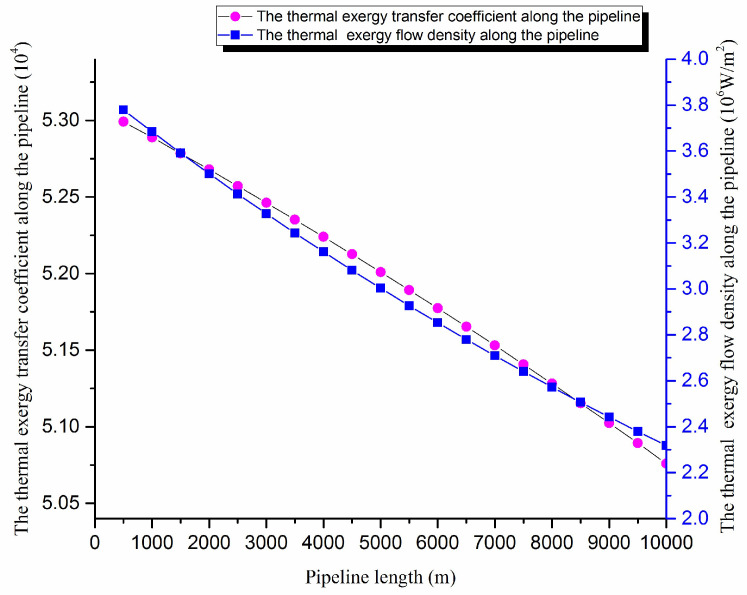
The thermal exergy transfer coefficient change curve and the thermal exergy flow density change curve of pipeline.

According to the crude oil basic physical properties and the operating parameters of the pipeline, the temperature field, the thermal exergy transfer coefficient and the thermal exergy flow density are calculated using the above equations. The thermal exergy transfer coefficient change curve and the thermal exergy flow density change curve along the pipeline are shown in Figure 6.

The axial temperature field of the pipe is gradually decreains. In Figure 6, the thermal exergy transfer coefficient and the thermal exergy flow density decrease along the transfer process, and the decreasing amplitude drops gradually because the temperature difference, which can drive the thermal exergy transfer, is greater at the beginning. With the advance of oil flow in the pipe, the temperature difference rises, and the heat transfer capacity of crude oil decreases gradually, so the thermal exergy transfer strength and ability are lessened.

1. Thermal exergy transfer analysis in the pipeline transportation process under different ambient temperature conditions

**Figure 7:**
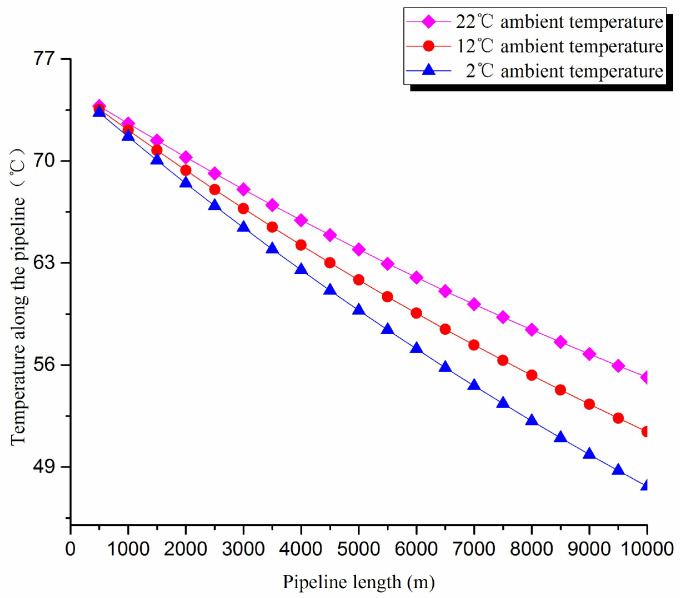
Pipe axial temperature field distribution curves under different ambient temperature conditions.

**Figure 8:**
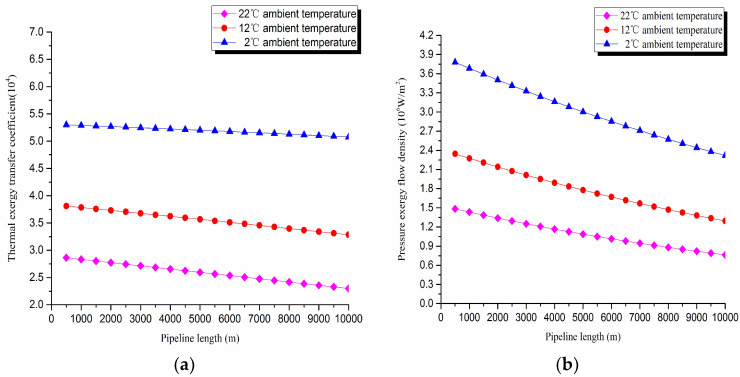
(**a**) Thermal exergy transfer coefficient change curves under different ambient temperature conditions. (**b**) Thermal exergy flow density change curves under different ambient temperature conditions.

The thermal exergy transfer law curves have been obtained under the working conditions of 2 °C, 12 °C, and 22 °C, as shown in Figure 7 and Figure 8.

From the thermal exergy transfer conditions under different ambient temperature, it can be seen that, when the ambient temperature is higher, the temperature drop is slower along the pipeline. In the initial stage of pipeline, the ambient temperature has little influence on the thermal exergy transfer coefficient. With the increase of transmission distance, the influence on thermal exergy transfer coefficient can be enhanced. However, the thermal exergy flow density change is slightly different from the thermal exergy transfer coefficient. The ambient temperature has great influence on thermal exergy flow density. With the increase of transmission distance, the influence becomes larger. In general, when the ambient temperature is higher, the thermal exergy transfer coefficient changes faster, and the thermal exergy flow density changes more slowly. However, the overall trend is decreasing. The thermal exergy transfer coefficient and the thermal exergy flow density are decreased with the rise of the ambient temperature. It can be explained as the increase of ambient temperature weakens the pipeline thermal exergy transfer phenomenon.

2. Thermal exergy transfer analysis in the pipeline transportation process under different outbound temperature conditions

**Figure 9:**
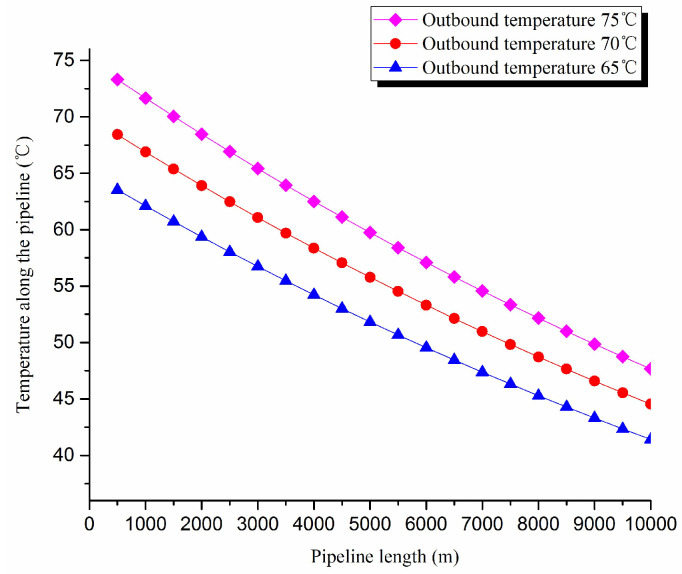
Pipe axial temperature field distribution curves under different outbound temperature conditions.

**Figure 10:**
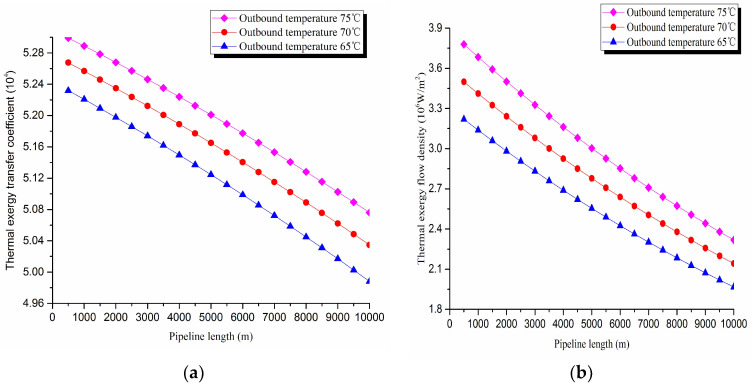
(**a**) Thermal exergy transfer coefficient change curves under different outbound temperature conditions. (**b**) Thermal exergy flow density change curves under different outbound temperature conditions.

The thermal exergy transfer law curves have been obtained under the working conditions of 65 °C, 70 °C and 75 °C, as shown in Figure 9 and Figure 10.

From the thermal exergy transfer conditions under different outbound temperature, it can be seen that, when the outbound temperature is higher, the temperature drop is faster along the pipeline. In the initial stage of pipeline, the outbound oil temperature has little influence on the thermal exergy transfer coefficient. With the increase of transmission distance, the influence on thermal exergy transfer coefficient can be enhanced. However, the thermal exergy flow density change is slightly different from the thermal exergy transfer coefficient. The outbound temperature has great influence on thermal exergy flow density at the beginning of the pipeline. With the increase of transmission distance, the influence is getting less. In general, when the outbound oil temperature is higher, the thermal exergy transfer coefficient changes faster, and the thermal exergy flow density changes more slowly. However, the overall trend is decreasing. The thermal exergy transfer coefficient and the thermal exergy flow density are increased with the increase of the ambient temperature. It can be explained as the rise of ambient temperature strengthens the pipeline thermal exergy transfer phenomenon.

3. Thermal exergy transfer analysis in the pipeline transportation process under different flow conditions

**Figure 11:**
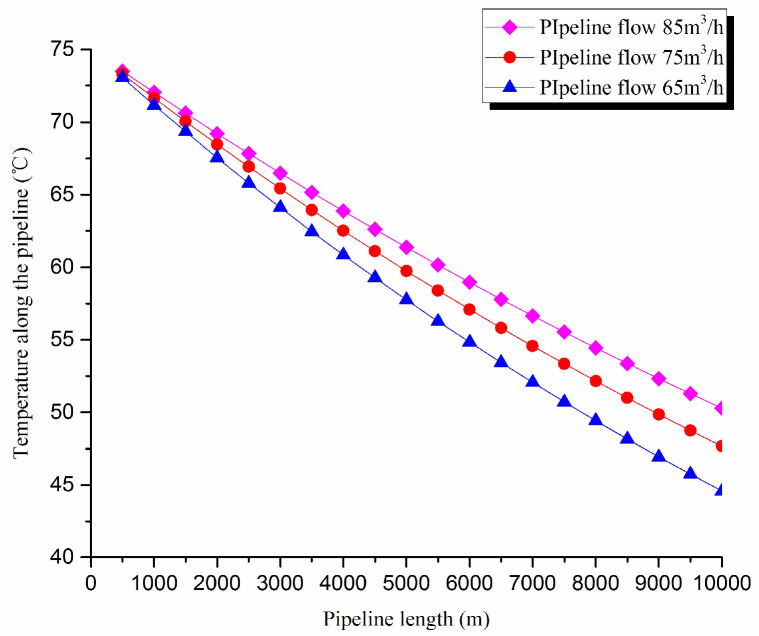
Pipe axial temperature field distribution curves under different flow conditions.

**Figure 12:**
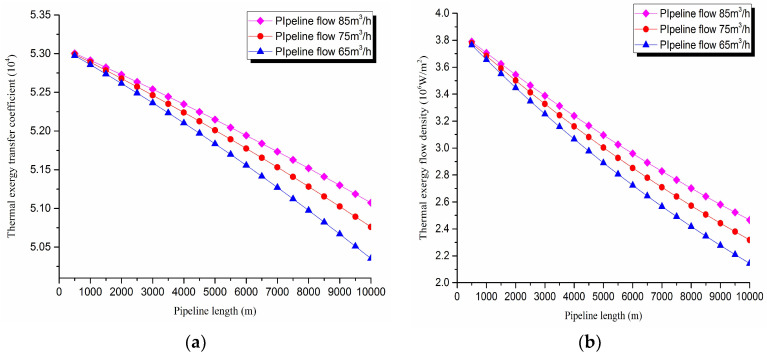
(**a**) Thermal exergy transfer coefficient change curves under different flow conditions. (**b**) Thermal exergy flow density change curves under different flow conditions.

The thermal exergy transfer law curves have been obtained under the working conditions of 65 m^3^/h, 75 m^3^/h, and 85 m^3^/h, as shown in Figure 11 and Figure 12.

From the thermal exergy transfer conditions under different flow, it can be seen that, when the flow is greater, the temperature drop is slower along the pipeline. In the initial stage of pipeline, the flow has little influence on the thermal exergy transfer coefficient. With the increase of transmission distance, the influence on thermal exergy transfer coefficient can be enhanced. In general, when the flow is greater, the thermal exergy transfer coefficient changes slower, and the thermal exergy flow density changes more slowly. However, the overall trend is decreasing. The thermal exergy transfer coefficient and the thermal exergy flow density are increased with the rise of the ambient temperature. It can be explained as the increase of ambient temperature strengthens the pipeline thermal exergy transfer phenomenon.

### 5.3. Analysis of Diffusion Exergy Transfer Influence Factors

**Figure 13:**
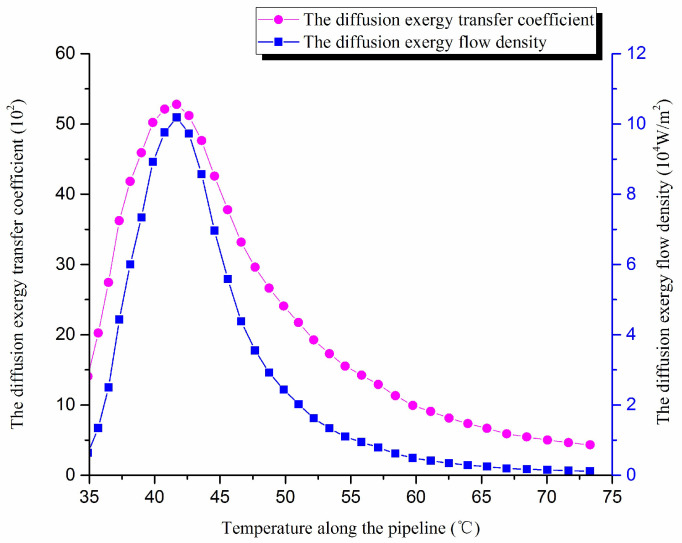
The diffusion exergy transfer coefficient change curve and the diffusion exergy flow density change curve of pipeline.

The pipeline length is 75 km, and the total heat transfer coefficient is 0.6 W/(M^2^ °C). The other pipeline operating parameters are the same above. The wax molecular concentration gradient, the diffusion exergy transfer coefficient and diffusion exergy flow density are calculated. The diffusion exergy transfer coefficient change curve and the diffusion exergy flow density change curve along the pipeline are shown in Figure 13:

As we can see, with the decrease of the oil temperature in the pipeline, the wax molecules diffusion rate increases first and then decreases, i.e., the wax molecular diffusion strength increases first and then decreases. The diffusion exergy transfer coefficient and the diffusion exergy flow density also increase first and then decrease. In the initial stage of pipeline, the oil temperature is high, and the wax molecular concentration gradient changes more slowly, which causes the diffusion exergy to change smoothly, the wax molecular diffusion rate to decrease, and the diffusion exergy transfer strength to reduce. With the decreasing temperature of crude oil, wax molecular concentration gradient changes gradually intensify, which causes the diffusion exergy transfer strength to also intensify. With the decrease of temperature, crude oil diffusion exergy changes greatly, which enhances the diffusion exergy transfer strength. With the flow forward, because of the reduction of temperature difference between the crude oil and the pipe wall, the wax molecules diffusion power is weaker, so the wax molecules diffusion rate is decreased, and the diffusion exergy transfer strength begins to weaken. Thus, in general, the diffusion exergy transfer strength and ability enhance first and then lessen with the decrease of temperature.

1. Diffusion exergy transfer analysis in the pipeline transportation process under different ambient temperature conditions

**Figure 14:**
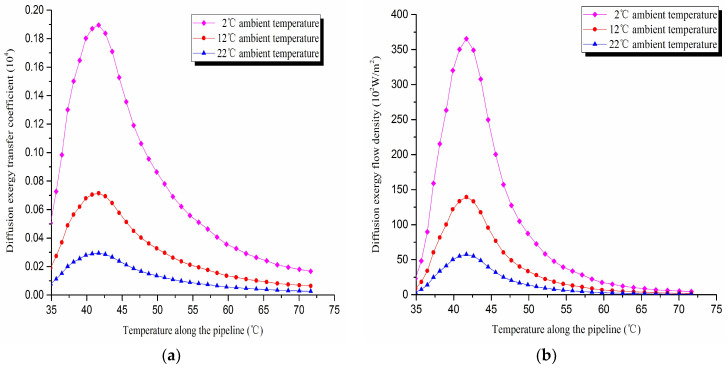
(**a**) Diffusion exergy transfer coefficient change curves under different ambient temperature conditions. (**b**) Diffusion exergy flow density change curves under different ambient temperature conditions.

The diffusion exergy transfer law curves have been obtained under the working conditions of 2 °C, 12 °C, and 22 °C in the pipeline transportation process, as shown in Figure 14.

From the diffusion exergy transfer conditions under different ambient temperature, it can be seen that the ambient temperature is higher, which causes the temperature difference between the oil flow and the tube wall to be small, and the wax molecules diffusion rate to decrease. The diffusion exergy transfer coefficient and the diffusion exergy flow density are all reduced. In the initial stage of pipeline, the rise of ambient temperature has little influence on the diffusion exergy transfer coefficient and the diffusion exergy flow density. Especially the change of the diffusion exergy transfer coefficient is also small at the early stage of the pipeline. With the increase of the transport distance, the influence of the two becomes increasingly important. In general, when the ambient temperature is higher, the diffusion exergy transfer coefficient and the diffusion exergy flow density change more slowly, but the overall trend is the same. It can be explained as the rise of ambient temperature weakens the pipeline diffusion exergy transfer phenomenon. The higher is the ambient temperature, the smaller is the wax molecules diffusion rate. This is due to the low ambient temperature, which leads to the less temperature difference between the oil flow and the tube wall. That causes the wax molecule diffusion rate to be small.

2. Diffusion exergy transfer analysis in the pipeline transportation process under different flow conditions

**Figure 15:**
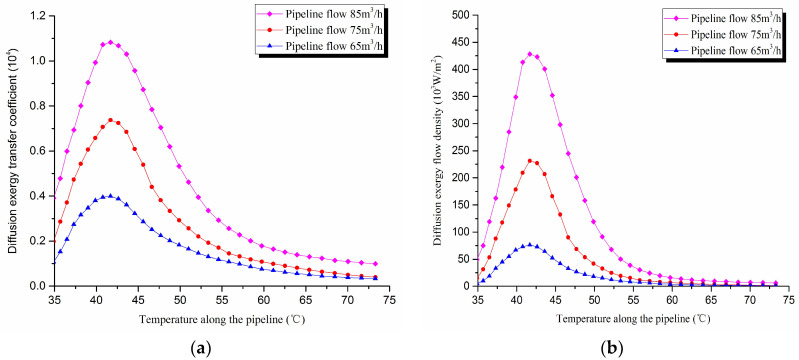
(**a**) Diffusion exergy transfer coefficient change curves under different flow conditions. (**b**) Diffusion exergy flow density change curves under different flow conditions.

The diffusion exergy transfer law curves have been obtained under the working conditions of 65 m^3^/h, 75 m^3^/h, and 85 m^3^/h, as shown in Figure 15.

It can be seen from the diffusion exergy transfer conditions under different flow, the pipeline flow is greater. The number of wax crystals molecules in the oil flow increases, and the wax molecular concentration gradient is higher. At the same time, the oil flow carries more energy, and the temperature gradient is higher between crude oil and the wall. Thus, when the wax molecular diffusion rate is large, the exergy transfer diffusion coefficient and the diffusion exergy transfer density are greater. In the initial stage of pipeline, the increase of flow has little effect on the diffusion exergy transfer coefficient and the diffusion exergy flow density. With the increase of the transmission distance, the impact of the two becomes increasingly important. In general, when the pipeline flow is higher, the diffusion exergy transfer coefficient and the diffusion exergy flow density change faster, but the overall trend is the same. It can be explained as the increase of pipeline flow enhances the pipeline diffusion exergy transfer.

## 6. Conclusions

Based on non-equilibrium thermodynamics, the calculation method of the entropy production rate for the waxy crude oil pipeline is obtained. Exergy conversion rules for crude oil pipeline process are calculated and analyzed. Considering the exergy transfer phenomenological equation and the exergy transfer dynamics fundamental equation, mathematical expressions in different forms of transfer coefficient and energy flow density are deduced for pipeline process. The change law and its influencing factors are further studied.

With the operation of pipeline, the transfer coefficient and flow density of thermal exergy and pressure exergy are decreased gradually. The increases of outbound temperature and outbound pressure enhance the thermal exergy transfer and pressure exergy transfer phenomenon. The increase of ambient temperature weakens the pipeline thermal exergy transfer. The increased pipeline flow strengthens the thermal exergy transfer phenomenon, but the pressure exergy transfer phenomenon is abated. 

With the decrease of oil temperature along the pipeline process, the diffusion exergy transfer coefficient and diffusion exergy flow density increase first and then decrease. The higher is the ambient temperature, the less temperature difference there is between the oil flow and the tube wall. The wax molecular diffusion rate is decreasing, so the diffusion exergy transfer coefficient and the diffusion exergy flow density are all reducing. It is indicated that a rise of ambient temperature weakens the pipeline thermal diffusion transfer. The higher is the ambient temperature, the slower are the changes of diffusion exergy transfer coefficient and diffusion exergy flow density. Conclusively, the increasing pipeline flow can intensify the diffusion exergy transfer phenomenon.

## Figures and Tables

**Table 1:** The physical parameters of crude oil and the operating parameters of pipeline.

Crude Oil Specific Heat Capacity	2.0 kJ/(kg·°C)	Pipeline Length	10 km
Crude oil Density	870 kg/m^3^	Pipeline diameter	219.1 mm
Crude oil dynamic viscosity	20.2 mPa·s (50·°C)	Pipeline external diameter	207.9 mm
Ambient temperature	2·°C	Pipeline start point temperature	75·°C
Environmental pressure	0.3 MPa	Pipeline start point pressure	5 MPa
Total heat transfer coefficient	2.6 W/(m^2^·°C)	Flow	0.0208 m^3^/s
Condensation point	28·°C	Average velocity	0.6137 m/s
Wax density	900 kg/m^3^	Wax content	26.29%

## Abbreviations

The following abbreviations are used in this manuscript:

*A*	an arbitrary variable (-)
*ε*	A density (-)
*c*	group element mass(kg)
*r*	component i mass generation rate in chemical reaction
*η*	mass fraction (-)
*E*	exergy (kJ)
*W*	flow velocity (m/s)
*L*	phenomenological coefficient (-)
*T*	temperature (K)
*V*	molar volume (m^3^)
*S*	entropy (kJ/K)
*T*	time (s)
*X*	intensive variable (-)
*K*	exergy transfer coefficient (-)
*j*	flow (-)
*e*	specific exergy (kJ/kg)
*p*	pressure (kPa)
*ρ*	density (kg/m^3^)
*π*	compressive stress tensor (-)
*τ*	shear force tensor (-)
Greek symbols	
*μ*	viscosity coefficient (kg/m∙s)
*δ*	unit tensor (-)
*Λ*	mass calculation coefficient of component in chemical reaction (-)
*σ*	entropy generation rate (-)
Subscripts	
0	initial state
*i*	component
*l*	length
Superscripts	
ex	exergy
